# Symptom, alexithymia and self-image outcomes of Mentalisation-based treatment for borderline personality disorder: a naturalistic study

**DOI:** 10.1186/s12888-018-1699-6

**Published:** 2018-06-11

**Authors:** J. Löf, D. Clinton, V. Kaldo, G. Rydén

**Affiliations:** 1 Stockholm Health Care Services, Stockholm County Council, Stockholm Psychiatry Southwest, Stockholm, Sweden; 2 Center for Psychiatry Research, Department of Clinical Neuroscience, Karolinska Institutet, Norra stationsgatan 69, 7 tr, 113 64 Stockholm, Sweden; 3 Institute for Eating Disorders, Oslo, Norway; 4 Stockholm Health Care Services, Stockholm County Council, Stockholm Psychiatry Southwest, Sweden, Center for Psychiatry Research, Department of Clinical Neuroscience, Karolinska Institutet, Stockholm, Sweden; 5 Child and Adolescent Psychiatry Clinic, Stockholm, Sweden

**Keywords:** Borderline personality disorder, Psychotherapy, Treatment outcome, Pragmatic clinical trials as topic, Mentalization-based treatment, Alexithymia

## Abstract

**Background:**

Mentalisation-based treatment (MBT) in borderline personality disorder (BPD) has a growing evidence base, but there is a lack of effectiveness and moderator studies. The present study examined the effectiveness of MBT in a naturalistic setting and explored psychiatric and psychological moderators of outcome.

**Method:**

Borderline and general psychiatric symptoms, suicidality, self-harm, alexithymia and self-image were measured in a group of BPD patients (*n* = 75) receiving MBT; assessments were made at baseline, and subsequently after 6, 12 and 18 months (when treatment ended). Borderline symptoms were the primary outcome variable.

**Results:**

Borderline symptoms improved significantly (*d* = 0.79, *p* < .001), as did general psychiatric symptoms, suicidality, self-harm, self-rated alexithymia and self-image. BPD severity or psychological moderators had no effect on outcome. Younger patients improved more on self-harm, although this could be explained by the fact that older patients had considerably lower baseline self-harm.

**Conclusions:**

MBT seems to be an effective treatment in a naturalistic setting for BPD patients. This study is one of the first studies of MBT showing that outcomes related to mentalisation, self-image and self-rated alexithymia improved. Initial symptom severity did not influence results indicating that MBT treatment is well adapted to patients with severe BPD symptoms.

**Trial registration:**

The study was retrospectively registered 25 September 2017 in the ClinicalTrials.gov PRS registry, no. NCT03295838.

## Background

Mentalisation-based treatment (MBT) [1, 2] posits that insecure attachment impairs the ability to reflect on one’s own and other’s inner mental states, especially in affectively stressful states, and that deficits in the ability to mentalise are conducive of psychopathology [3, 4]. Treatment is relational and focuses on better understanding and use of mentalising skills in order to promote affect tolerance and the ability to think flexibly while experiencing intense affect, rather than using self-harm or other kinds of impulsive behaviour to regulate affect states. The efficacy of MBT in the treatment of borderline personality disorder (BPD) has been demonstrated in three randomised controlled trials (RCTs) [1, 2, 5]. Two long-term follow-up studies suggest that the effects of MBT are lasting [6, 7], and improved mentalising has been shown in two studies with adolescents with borderline problems [5, 8], but so far not in relation to adult patients.

Although these studies provide important evidence concerning the therapeutic potential of MBT, a number of important problems remain. Few studies have been conducted outside the UK, where MBT was developed. What’s more, efficacy studies have for the most part been carried out by the researchers who designed and developed MBT, which leaves these studies open to criticisms of bias and allegiance effects. A randomised controlled trial in Denmark carried out by an independent group of researchers [9] compared MBT with supportive psychodynamic group psychotherapy at the end of treatment 2 years after intake. Both treatment arms showed significant improvements, but MBT was superior to the control treatment only in regard to patients’ general assessment of functioning (GAF). However, GAF ratings were made by therapists who were not blind to treatment arm, which could have compromised validity. There was also a skewed allocation to treatment conditions in the Danish study and a general lack of adherence to the MBT treatment manual, along with a lack of expert supervision in MBT.

There has been a lack of naturalistic studies examining the effectiveness of MBT as it is implemented in community-based psychiatric settings, which also limits the evidence base. Although two studies have employed naturalistic designs and demonstrated good effectiveness of MBT on BPD symptoms and functioning [10, 11] they were not community-based. Moreover, in one of these studies [11] it is not clear how BPD diagnosis was established, nor whether diagnoses were valid and reliable since no information was provided on possible exclusion criteria.

Bateman and Fonagy have performed further analyses of their own data and found that comorbidity of BPD with other personality disorders is a factor necessitating MBT rather than supportive treatment [12]. Their study raises the question of systematic treatment selection, which in a recent study has been shown to be effective for psychodynamic therapy (PDT) [13]. Systematic treatment selection would allow for the identification of lower mentalisation abilities, as well as more personality and interpersonal problems, indicating a need for mentalisation-based interventions. In particular, hypermentalising, negatively biased overinterpretation of interpersonal situations, has been shown to be connected to the severity of borderline problems and may possibly mediate change in MBT [14, 15]. Alexithymia has been shown to be highly related to BPD [16, 17]. The concept can be defined as difficulties in identifying and distinguishing feelings from bodily sensations and problems in expressing these feelings to others. It is considered to be an aspect of affective mentalisation (i.e. of the self) [18]. Negative self-image has been shown to moderate change in PDT in that baseline severity is related to greater symptom reduction [19]. In line with this result and the theory behind the treatment, it is possible that low mentalisation ability could be related to suitability for MBT. Evidence was, however, considered to be too sparse to formulate a hypothesis with regard to what could moderate effects of MBT. We extended moderating factors to be studied from comorbidity used in Bateman and Fonagy’s analysis [12] to also include self-rated alexithymia, self-image and attachment style. Self-rated alexithymia and self-image was also examined as an outcome of treatment.

### Aims of the study

The purpose of the present study was firstly to examine naturalistic outcomes (i.e. borderline and general psychiatric symptoms, suicidality, self-harm, self-rated alexithymia and self-image) in an implementation of MBT for BPD in a Swedish psychiatric outpatient setting, and secondly to study patient baseline correlates of effectiveness (moderators). We expected improvements on all outcomes whereas the study of moderators was exploratory.

## Methods

### Design and setting

BPD patients participating in the MBT outpatient programme (a part of the standard psychiatric services provided by Stockholm Regional Health Care Services, Psychiatry Southwest) were assessed at baseline on all measures and subsequently after 6, 12 and 18 months on primary and secondary outcome measures.

### Patients

Patients were recruited from the MBT outpatient programme for BPD at Huddinge University Hospital. The programme is community-based and publically financed, being operated within the psychiatric services of Stockholm Regional Council. Prospective patients with a probable BPD diagnosis were referred from psychiatric clinics and primary care units in the metropolitan Stockholm area. To be included, BPD diagnosis was confirmed by SCID-II interview and the Zanarini Rating Scale for Borderline Personality Disorder (ZAN-BPD) interview by the MBT-team therapists, including consensus discussion using DSM-IV and ICD-10 criteria. Exclusion criteria were: IQ < 85, psychotic disorder other than schizotypal personality disorder, acute/temporary psychosis, previously diagnosed autism-spectrum disorder, bipolar disorder type I and severe eating or substance use disorder. IQ was screened using three subtests of the Wechsler Adult Intelligence Scale-III (WAIS-III), and, when indicated by low screening scores, the full test. All patients referred between 2007 and 02-01 and 2012–05-30 were eligible for inclusion. All patients signed written informed consent forms to participate in the research.

### Therapists

MBT therapists were 1–2 psychiatrists, 2–4 clinical psychologists and, for parts of the study period, a psychiatric nurse. All therapists were trained at Anna Freud Centre, basic and advanced courses. During the study period there were 1–2 supervisory days yearly with A. Bateman where adherence to the treatment model was reviewed. There was also weekly general supervision.

### Treatment

MBT was conducted according to the treatment manual developed by Bateman & Fonagy [20]. Patients were offered individual sessions with a psychotherapist and group sessions with 6–8 participants and 1–2 group therapists for 18 months. An introductory psycho-educational component (9–12 sessions) was also offered focusing on explicit mentalising skills (i.e. understanding one’s own or others’ intentions). Mentalising and the treatment structure and focus were explained through short presentations and exercises. Group and individual MBT focused on implicit mentalising towards self and others. From August 2005 the programme consisted of 1 weekly individual session, 2 weekly MBT group sessions and 2 weekly expressive group sessions (*N* = 8 patients, 11% of sample). Expressive sessions were comprised of weekly writing and art sessions. From August 2008 the programme was reduced to one individual and two MBT group sessions (*N* = 13 patients, 17% of sample). In June 2009 it was changed to one individual and one group session (*N* = 54 patients, 72% of sample). Therapy sessions were videotaped and reviewed by the entire team weekly to monitor adherence. Psychopharmacological treatment was provided by the team’s psychiatrist. Pharmacological treatments was prescribed for comorbid disorders using regional guidelines and not for the borderline condition per se. The most common disorders in this respect was depressive episodes, ADHD, and sleeping disorders. Anxiety symptoms were primarily seen as part of the borderline personality disorder. Serotonine reuptake inhibitors (SSRIs) was by far the most common medication used. Methylphenidate and other central stimulants as well as lithium, antipsychotic and antiepileptic medication were also prescribed. Bensodiazepines were prescribed with great care and restriction. Following Bateman & Fonagy [2], treatment completion was considered to be 12 months of treatment or more. After completion of the treatment programme individually tailored follow-up was provided if needed; this could consist of psychiatrist visits, group or individual therapy sessions.

### Measures

#### Primary outcome

Key psychiatric and borderline symptomatology as measured by the *Karolinska Borderline And Symptoms Scales* (KABOSS-S) [21] was the primary outcome measure. The KABOSS-S consists of three general symptom subscales (Depression, Anxiety, Obsessive-compulsive Symptoms) derived from the Comprehensive Psychopathological Self-rating Scale for Affective Syndromes, as well as one specific borderline subscale consisting of the items “mood swings”, “ability to understand own emotions”, “self-control”, “self-soothing”, “feelings of abandonment”, “feelings of emptiness”, “self-image” and “reality presence”. Each item is scored on a Likert scale from 0 (“no presence”) to 6 (“severe”). KABOSS-S general symptom subscales have been validated through high correlations with clinician interview ratings of depression (*r* = .83) and anxiety (*r* = .76). The borderline symptom subscale could identify BPD patients and was highly internally consistent (Cronbach’s internal consistency of 0.90) [22].

#### Secondary outcomes

Suicidality was measured by the *Suicide Assessment Scale, Self-Report* (SUAS-S) [23], which covers factors known to influence suicide risk, such as affect, bodily states, control and coping, emotional reactivity, as well as suicidal thoughts and behaviour. Ratings of SUAS-S in a cohort of inpatients has been shown to identify those who attempt suicide [24]. Validity of the SUAS-S self-report version has been reported with a highly significant correlation (ρ = .82) with the interview version of the SUAS and with the Montgomery Åsberg Depression Rating Scale (ρ = .78) [24]. General psychiatric symptoms were measured using the *Symptom Checklist-90 Revised* (SCL-90-R) [25]. Self-harm was measured by the *Deliberate Self-Harm Inventory-9* (DSHI-9) [26]. This measure was, however, introduced halfway through the study period (*N* = 42). The DSHI-9 was not normally distributed; 38% of patients had a baseline score of zero (e.g. no self-harm behaviour).

The *Toronto Alexithymia Scale-20* (TAS-20) [27] was used to measure self-rated alexithymia. It comprises 20 items divided into three subscales: Difficulty Identifying Feelings, Difficulty Expressing Feelings and Externally Oriented Thinking. TAS-20 was used to measure self-rated affective mentalisation [16]. Test-retest reliability has been reported as 0.77 [27].

Self-image was assessed using *Structural Analysis of Social Behavior* (SASB) [28]. SASB is based on a circumplex model, measuring self-image and interpersonal interactions in relation to three interpersonal “surfaces” (i.e. actions of others, reactions to others and the introject, or what can be called the self-image. The third surface (self-image) was used in the present study, which comprises eight clusters of self-image: 1) Autonomy; 2) Self-affirmation; 3) Active self-love; 4) Self-protection; 5) Self-control; 6) Self-blame; 7) Self-attack; and 8) Self-neglect. Since there were high positive correlations between clusters 2–4 and clusters 6–8, suggesting overlapping data, the two sets of clusters were merged into two variables, “Self-love” (clusters 2–4) and “Self-hate” (clusters 6–8).

#### Moderator, diagnostic and background measures

We used measures of alexithymia and self-image at baseline as moderator measures in addition to using them to measure outcomes over time. Attachment style at baseline was also explored as a moderator and was assessed using the *Relationship Questionnaire* (RQ), a 4-item self-report measure yielding scores on “Avoidant attachment”, “Secure Attachment”, “Preoccupied attachment” and “Fearful attachment” [29]. SCID-II [30] was used to assess DSM-IV Axis-II disorders. The MINI-International Neuropsychiatric Interview assessed DSM-IV Axis-I disorders [31], and the ZAN-BPD structured interview [32] was used to measure borderline symptom severity. Comorbidity and borderline severity at baseline were used as moderator measures. ADHD diagnoses were based on case notes. Patients also completed a 27-item demographic questionnaire covering background, previous treatment and trauma.

#### Statistical analysis

A linear mixed model (LMM) was used to evaluate change over all time-points (baseline and 6, 12, and 18 months after treatment start) on primary and secondary measures. We analysed data according to the “intent to treat” principle. Missing data for assessment points during and after treatment was 47–74%. Factors and covariates included in LMM analyses were Axis I and Axis II comorbidity (number of diagnoses), ZAN-BPD baseline score, RQ scales, SASB subscales and TAS-20. We assumed that data were missing at random. We entered age as a factor due to higher baseline self-harm scores for younger patients. Due to childhood sexual trauma being correlated with several severity indicators, it was also entered as a factor. We attempted to control for differences in treatment format by entering substance use disorder and antisocial PD as factors, since they were overrepresented in one treatment format. More Axis-I comorbid disorders at baseline were related to higher rates of missing data; within the Axis-I comorbidity factor, patients with eating disorders were less likely to complete self-reports. To control for this, we entered eating disorder diagnosis as a factor. All non-parametric scores were dichotomized at the median (Axis I: 0–2 = 0; 3 + =1, Axis II: 0–1 = 0, 2 + =1; ZAN-BPD: 1–16 = 0, 17 + =1; RQ A: 1–3 = 0, 4 + =1, RQ B: 1–2 = 0, 3 + =1, RQ C: 1–4 = 0, 5 + =1, RQ D: 1–5 = 0, 6 + =1; age 19–28 years = 0, 29–51 years = 1). Subject was entered as random, all others as fixed factors. AR(1) was chosen as the co-variance matrix and estimation was based on restricted maximum likelihood. Non-significant factors were dropped. Fixed factors at baseline that contributed to the model but had no effect on outcome (i.e. no interaction with time) are not reported but results are available upon request. Effect sizes for pre- and post-treatment differences were calculated from estimated marginal means and standard deviations derived from standard errors. For primary measures, we conducted tests using each time point to analyse when change occurred. We analysed dropouts and patients who did not provide self-reports in order to examine if baseline primary and secondary symptom scores or BPD severity was related to dropout or not providing self-reports. All statistical analyses were conducted using SPSS 17.0 [33].

## Results

### Patient characteristics

The sample had a mean age of 30.4 years (SD = 7.7, range 19–51). Background data and comorbidity are provided in Table 1. The 2-session treatment format had significantly higher levels of patients working or studying, fewer patients on sickness benefit, fewer patients with antisocial PD and fewer patients with a substance use disorder. Patients with high self-harm scores on the DSHI-9 were younger and more often had no tertiary education. Victims of childhood sexual trauma had significantly higher borderline symptoms on the KABOSS-S and suicidal ideation on the SUAS-S. They were also more likely not to be working or studying, to have a depressive disorder and PTSD. Victims of rape after 15 years of age were more likely to be female, have a substance abuse disorder and to belong to the highly comorbid Axis I-group. Victims of physical abuse were more likely to have ADHD.

**Table 1:** Background data of included patients

Variable	Percentage of all patients
Sociodemographic variables	
Sex, female	89.3%
Married/cohabiting	37.3%
Have children	28.0%
Working/studying	42.7%
Tertiary Education^a^	30.7%
Sickness benefit	48.0%
Trauma	
Sexual abuse < 15 yrs	42.9%
Physical abuse < 15 yrs	52.9%
Rape 15 yrs.>	45.9%
Trauma (any)	76.1%
Loss of parent < 18 yrs	6.7%
Axis I diagnosis	
Depressive disorder (any)	58.7%
Bipolar disorder (any)	2.7%
PTSD	25.3%
Anxiety disorder (any except PTSD)	68.0%
Psychotic disorder (any)	4.0%
Substance Abuse disorder (any)	20.0%
Eating disorder (any)	22.7%
ADHD	24.0%
Axis II diagnosis	
Paranoid PD	20.6%
Schizoid PD	0
Schizotypal PD	5.9%
Cluster A PD, total	26.5%
Histrionic PD	2.9%
Narcissistic PD	4.4%
Borderline PD	100%
Antisocial PD	7.4%
Cluster B PD other than BPD, total	13.2%
Avoidant PD	41.2%
Dependent PD	20.6%
Obsessive-Compulsive PD	16.2%
Cluster C PD, total	57.4%

^a^Tertiary education = post-high school/gymnasium education, i.e. university or community college

The mean number of personality disorder diagnoses other than BPD was 1.21 (SD = 1.16, range 0–4), the mean number of Axis-I disorders was 3.10 (SD = 1.86, range 0–7). Borderline symptom severity on the ZAN-BPD had a mean of 16.8 (SD = 4.94). Patients with high BPD severity on the ZAN-BPD were more likely to belong to the highly comorbid Axis-I group and to belong to the highly fearfully attached group. Axis-II comorbidity was connected to belonging to the low secure attachment group.

### Treatment dropout and attrition

**Fig. 1:**
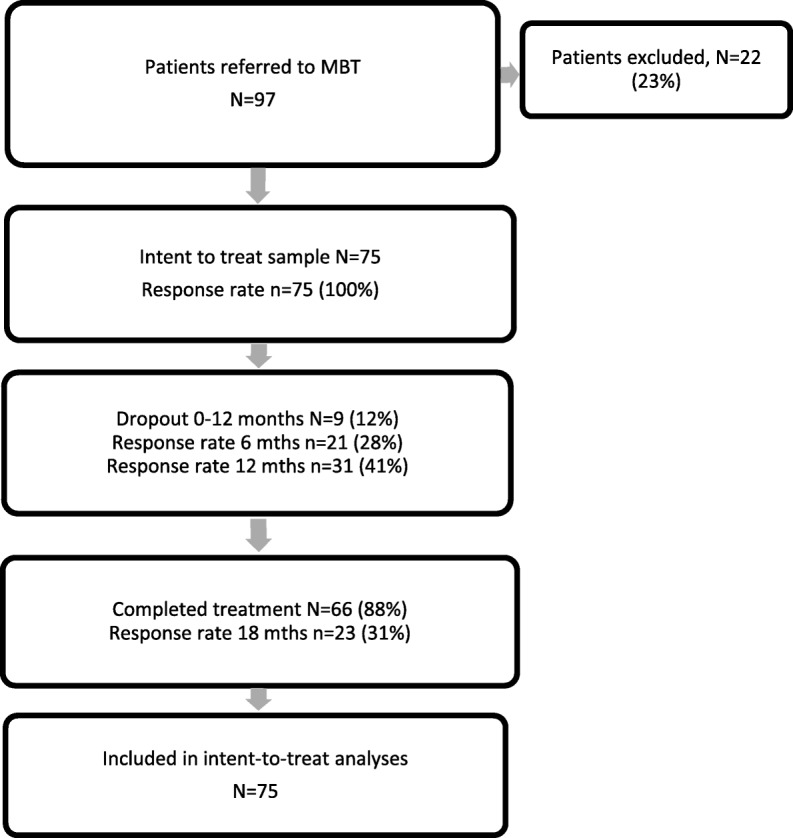
Patient progression through the MBT programme and response rate to primary outcome self-report

Patient flow is illustrated in Fig. 1. Patients who completed treatment had a mean of 17.0 months of treatment (SD = 1.83), while dropouts completed a mean of 5.7 months of treatment (SD = 3.94); 74% of patients that completed at least 12 months of treatment and 65% of all patients completed the full 18 months of the programme. Dropouts did not differ significantly at baseline on any of the primary or secondary symptom measures or on any indicator of BPD severity (Axis I or Axis II comorbidity, ZAN-BPD scores).

Patients who did not respond to self-reports did not differ on any of the primary or secondary symptom measures or on any indicator of BPD severity (Axis I & II comorbidity, ZAN-BPD scores). Patients with no self-report data at follow-up were not more likely to dropout.

### Primary outcome

Borderline symptoms on the KABOSS-S improved significantly over time (see Table 2). Effect size for the borderline symptom subscale (*d* = .84, *p* < .001) was similar to the general symptom subscales (*d* = .76, *p* < .001). When analysing each time-point, the 12-month *r* score comparison with baseline scores was significant (*d* = .52, *p* < .01), but not the 6 month comparison with baseline scores. Significance remained at this level when checking for multiple comparisons using Bonferroni correction. The comparison between 12 and 18-month data showed further improvement (*d* = .26, *p* < .05). However, when adjusting for multiple comparisons with the Bonferroni test, significance for the 12 to 18-month comparison dropped (*p* = .14). Thus, change in borderline symptoms seemed to happen primarily during the first year as well as to a lesser extent between 12 and 18 months.

**Table 2:** Outcomes of MBT. Linear mixed model (0, 6, 12 and 18 months, REML estimation)

Measured Variable	Baseline (Range of N 72–75)	6 months (Range of N 21)	12 months (Range of N 23–31)	18 months (Range of N 22–24)	Regression data	
	M (SD)	M (SD)	M (SD)	M (SD)	t	d
Symptom Questionnaires						
KABOSS-S	86,6 (21,0)	73,1 (27,9)	66,2 (26,0)	54,5 (28,0)	5,10^***^	0,79
SCL-90 GSI	1,83 (0,64)	1,47 (0,57)	1,46 (0,69)	1,26 (0,79)	3,88^***^	0,58
Suicidality						
SUAS-S	40,3 (10,9)	35,1 (15,1)	31,0 (14,4)	25,1 (14,9)	5,58^***^	0,62
Alexithymia						
TAS-20 DIF	23,1 (4,84)	22,6 (5,45)	19,2 (6,99)	19,8 (7,03)	3,33^**^	0,52
TAS-20 DDF	16,0 (4,66)	14,2 (4,00)	14,8 (4,86)	14,1 (5,44)	2,26^*^	0,29
TAS-20 EOT	18,8 (4,97)	16,8 (4,90)	18,4 (5,30)	16,3 (4,85)	2,05^*^	0,25
Self-image						
SASB Autonomy	28,5 (14,7)	31,0 (16,1)	28,3 (18,2)	34,8 (15,3)	3,00^**^	0,39
SASB Self-love	26,6 (14,8)	40,0 (21,1)	36,4 (21,3)	50,9 (21,2)	7,96^***^	1,00
SASB Self-hate	55,5 (16,4)	41,4 (21,2)	45,9 (21,5)	32,2 (22,8)	5,39^***^	0,75
SASB Self-control	41,7 (20,6)	54,8 (19,2)	54,3 (20,7)	57,8 (18,5)	3,73^***^	0,54

^*^ = *p* < .05, ^**^ = *p* < .01, ^***^p < .001. *M* mean, *SD* standard deviation, *t* t-value for regression, *d* Cohen’s d calculated from marginal means. *KABOSS-S* borderline symptoms, *SUAS-S* suicidality, *SCL-90* general psychiatric symptoms, *TAS-20* Alexithymia, *DIF* “Difficulties Identifying Feelings”, *DDF* “Difficulties Describing Feelings”, *EOT* “Externally Oriented Thinking”, *SASB* Self-image. Self-love is the mean of clusters 2–4; Self-hate the mean of clusters 6–8

### Secondary outcomes

Suicidality on the SUAS-S and general psychiatric symptoms on the SCL-90 improved over time. Self-rated alexithymia on the TAS-20 improved on all subscales, although the “Difficulty Identifying Feelings” subscale had a slightly higher effect size than the other the subscales. On measures of SASB self-image, Autonomy, Self-love, Self-hate and Self-control all improved over time. Self-harm on the DSHI-9 also improved over time (baseline *n* = 42, M = 11.5, SD = 14.2; 6 months *n* = 22, M = 9.50, SD = 11.4; 12 months *n* = 23, M = 6.57, SD = 9.76; 18 months *n* = 22, M = 4.62, SD = 7.34; regression data β = 6.34, SE = 2.44, Wald Z = 6.79, *p* < .01, d = 0.49).

### Moderation and interaction

There were no interactions with change in primary and secondary outcomes over time or with fixed factors (Axis I and Axis II comorbidity, borderline severity on the ZAN-BPD and psychological moderators; TAS-20, SASB, RQ). Since age was highly correlated with self-harm, age was also added as a moderator. Age was found to moderate change in self-harm on the DSHI-9; patients younger than 39 years of age improved more over time on self-harm (β = 7.39, SE = 3.44, Wald = 4.63, *p* < .05). When the interaction was entered, improvement over time only remained significant for the treatment start to 12 months comparison (β = − 3.00, SE = 1.45, Wald = 4.26, p < .05). This could be explained by the fact that for older patients DSHI-9 scores were close to zero at 12 months, i.e. a floor effect (M = .25, SD = .50). The moderating interaction of age with time could have been influenced by the higher rates of baseline self-harm in younger patients (18–24 years: 91%, 25–30 years 60%, 31–39 years 54% and 40 years or older 38%).

## Discussion

Mentalisation-based treatment was associated with improvements in borderline and general psychiatric symptoms as well as suicidality, self-harm, self-rated alexithymia and self-image. The dropout rate of 12% was low compared to a systematic review of PD treatments, but similar to other MBT trials [2, 11, 34]. Effect sizes for primary and secondary symptom outcomes were in the range of *d* = 0.49–0.79. If we compare general psychiatric symptom effect size (*d* = 0.58), it was similar to that found by Jorgensen and co-workers’ study of the MBT outpatient programme (*d* = 0.61) [9] but less than the original study (*d* = 1.04) [2]. The naturalistic study by Kvarstein and colleagues showed a higher effect size (*d* = 1.05) [11], but treatment length in that study was 3 years rather than 18 months. Longer follow-up periods of our treatment programme are planned and could demonstrate that higher effects are dependent on further treatment as in the Kvarstein et al. study, or alternately that there could be a stronger delayed effect of the 18-month programme having an effect over time. According to a meta-analysis of comparable treatments, some further improvement in general psychiatric symptoms during follow-up is to be expected [35]. Higher effect sizes in RCT studies of psychotherapy compared to naturalistic studies have been observed for some outcomes in a meta-analysis, and an effect of treatment fidelity has been found [35]. The explanation most applicable to this study is the relative lack of expert supervision compared to what is typical of RCT:s [36]. Supervision has been shown to have an effect on treatment adherence [37].

The central point aspect of the MBT model is that difficulties in affect regulation in BPD are thought to be related problems of mentalisation. An important result of the present study was therefore the finding that measures of self-reported alexithymia improved over time, suggesting that treatment improved patients’ capacity to identify feelings. Alexithymia has been shown to be associated with self-reported reflective functioning [38] as well as highly related to reflective functioning scored on the Adult Attachment Interview in depressed patients [39]. In the present study patients undergoing MBT appeared to increase their capability to identify emotions and direct their thinking towards internal states. A more nuanced and differentiated awareness of their own emotional states could be involved in the reduction of borderline symptoms and self-harm, although mediator studies would be needed to confirm this. Other modes of assessing alexithymia than self-ratings would also be needed. We did not measure other aspects of mentalisation such as affective mentalisation of others or cognitive mentalisation of self and others. Since self-reported reflective functioning using the Reflecting Functionning Questionnaire (RFQ) [38, 40], a more specific instrument for assessing a key aspect of mentalisation, has been shown to have moderate to high correlations with self-rated alexithymia, it will be important for future studies to use the RFQ.

We found that patients had a very negative self-image at the start of treatment. This reflects the mentalising conceptualisation of intensive negative self-representations in BPD due to trauma, neglect and poor mirroring (e.g. “the alien self”). It can also be found in psychodynamic transference-based therapy, where negative object representations are considered to be internalised and involved in projections, as well as in dialectical behaviour therapy where a lack of validation by others is thought to lead to a lack of self-validation. Self-image has been shown to be highly related to BPD, especially on the “Affiliation axis”, i.e. patient have low self-love and high self-hate [41–44]. We saw improved self-image on all aspects of the SASB after MBT and effect sizes were highest in relation to greater Self-love and Self-protection and less Self-attack. This can be seen as a preliminary confirmation of central aspects of the MBT model, such as affect focus and mentalised affectivity, improving the patient’s internal working model of secure attachment representations (i.e. capability of self-soothing and self-compassion), as well as reducing the intensity of negative self-representations. While other evidence-based BPD treatments could plausibly also result in improved self-image, this has only been studied in one DBT trial [44]. Self-image has also been shown to improve in a naturalistic study of a psychodynamic therapeutic community including borderline patients [45]. Taken together, our results and those of others suggest that a mentalising approach on the part of therapists may be especially conducive to the internalisation of self-compassion.

Moderator effects were few and borderline severity did not influence results. Severe patients improved as much as less severe patients, even though they had worse symptoms at baseline. This is in line with Bateman and Fonagy’s re-analysis [12] of their RCT of the outpatient programme where MBT was concluded to be effective with the most severe patients. A moderator effect for age, however, was found on self-harm on the DSHI-9; patients younger than 39 years improved more on self-harm. The interaction might in part be due to younger patients self-harming more than older ones and thus having something that could be treated in this domain. Another possible interpretation is that maturation is connected to less impulsivity, a process that treatment could reinforce.

There are several limitations to the present study. Firstly, we had no control group. Thus, we cannot ascertain if the observed changes are due to MBT, natural improvements in the course of BPD, measures being biased by social desirability (possibly exaggerated by these patients’ tendency to show ‘apparent competence’ in some situations), or an effect that could have been achieved by a less extensive treatment type of treatment. Secondly, response rates for self-reports at follow-up were generally low. Linear mixed modelling, however, allows for retention of more data than traditional statistical analyses. Nevertheless our low self-report response rate was hard to analyse, making it difficult to rule out responder biases. Thirdly, the treatment format changed during the study period. This means the treatment received differed in intensity between patients. There were some indications that patients in the more intensive treatment formats were more low-functioning. This could possibly be due to a programme of MBT for substance abuse starting in Stockholm midway through our study period, which may have resulted in some patients being referred there. Neither of these severity indicators nor treatment format had an effect on outcomes, although power to detect these differences was limited. Fourthly, alexithymia, self-image and attachment were measured only through self-report; other modes of measurement such as interview or observer ratings might capture these dimensions more objectively. Fifthly, the MBT team in Huddinge, being the first unit in Sweden to implement the model, had irregular access to specialised MBT supervision and no outside video adherence ratings. Future studies would need to implement a more stringent control of the treatment.

## Conclusions

The present study strengthens the case that MBT is an effective treatment for borderline patients that can be implemented in routine mental health care. The study adds to the growing evidence base on MBT by showing changes in affective mentalisation and self-image during treatment, and moves into the territory of testing what happens with patients’ psychological functioning during MBT, which some studies on adolescents have started to do [8, 15]. More research that is better powered to detect moderator effects is needed in order to better test which factors are important in MBT. Randomised controlled trials comparing MBT with other bona fide PD treatments are also needed. A promising future direction of research is studying systematic treatment selection, which has been shown to be able to improve treatment results over randomisation for PDT [13]. In earlier research both high psychological mindedness, a concept akin to cognitive mentalisation, and low alexithymia have been found to favour response to PDT [46, 47]. MBT, on the other hand, was developed to be effective especially for patients with mentalising difficulties. Our finding that patients in MBT improve regardless of initial severity suggests that MBT is effective for BPD patients seeking specialised psychiatric care.

## Abbreviations

BPD: Borderline personality disorder
DSHI-9: Deliberate Self-Harm Inventory-9
GAF: Global assessment of functioning
GSI: Global severity index
LMM: Linear mixed model
MBT: Mentalisation-Based Treatment
PDT: Psychodynamic therapy
RCT: Randomised controlled trial
RQ: The relationship questionnaire
SASB: Structural analysis of social behavior
SCID: Structured Clinical Interview for DSM-IV
SCL-90-R: Symptom Checklist-90 – Revised
SUAS-S: Suicide Assessment Scale, Self-Report
TAS-20: Toronto Alexithymia Scale-20
ZAN-BPD: Zanarini rating scale for borderline personality disorder
